# VEGF promotes gastric cancer development by upregulating CRMP4

**DOI:** 10.18632/oncotarget.7717

**Published:** 2016-02-25

**Authors:** Sile Chen, Xinhua Zhang, Jianjun Peng, Ertao Zhai, Yulong He, Hui Wu, Chuangqi Chen, Jinping Ma, Zhao Wang, Shirong Cai

**Affiliations:** ^1^ Department of Gastrointestinal Surgery Center, The First Affiliated Hospital of Sun Yat-sen University, Guangzhou 510080, Guangdong Province, China

**Keywords:** gastric cancer, angiogenesis, immunohistochemistry, ERK/AKT pathway

## Abstract

This study aimed to investigate the precise role of CRMP4 in gastric tumor growth and patient survival. The mRNA and protein expression levels of CRMP4, VEGF and VEGFR2 were validated by qRT-PCR and immunohistochemistry. We investigated the effects on tumor growth of overexpression and knockdown of CRMP4 both *in vitro* and *in vivo* by constructing stable gastric cell lines using lentiviral-mediated transduction and shRNA interference-mediated knockdown of CRMP4 expression. We further validated the role of the ERK/AKT signaling pathways in VEGF and CRMP4 expression using ERK and PI3K inhibitors. Increased expression of VEGF and CRMP4 were observed in gastric cancer tissues compared with tumor-adjacent tissue. We found that higher CRPM4 expression was associated with lymph node metastasis, TNM stage, tumor differentiation and poorer prognosis in gastric cancer patients. In HGC27 and SGC7901 gastric cancer cells, VEGF upregulated CRMP4 in time and dose-dependent manners. Overexpression of CRMP4 increased cell proliferation, migration and invasion, whereas knockdown of CRMP4 expression had opposite effects. VEGF activated CRMP4 expression in gastric cancer cells, and this effect was significantly inhibited by MAPK and PI3K inhibitors (PD98059 and LY294002). In mice, CRMP4 overexpression also resulted in increased tumor growth. These results suggest that increased CRMP4 expression mediated by the activation of VEGF signaling facilitates gastric tumor growth and metastasis, which may have clinical implications associated with a reduced survival rate in gastric cancer patients.

## INTRODUCTION

Gastric cancer is the third most common malignant disease and the leading cause of high morbidity and mortality in cancer patients worldwide [1, 2]. A recent report investigating the incidence of cancer and associated mortality in China showed that gastric cancer ranked second among all malignancies, with nearly 1 million cases and cancer-related deaths [3].

Angiogenesis is necessary for the growth and metastasis of solid tumors, and vascular endothelial growth factor (VEGF) is the most potent angiogenic mediator [4–9]. A critical pillar of angiogenesis is the interaction of the VEGF family of proangiogenic cytokines and their respective receptors. VEGFR2 expression is typically limited to vessel endothelial cells, and is widely considered as the main receptor driving angiogenesis [10]. Moreover, VEGF and VEGF receptors, particularly VEGF receptor 2 (VEGFR2/Flk-1), are considered to constitute the key signaling system regulating endothelial cell proliferation and migration [10, 11]. Clinical trials have shown that administration of anti-VEGF antibody combined with chemotherapy significantly prolonged the survival of colorectal cancer patients [12] and progression-free survival in gastric cancer patients [13].

Collapsin response mediator proteins (CRMPs) have been reported to be associated with proliferation, apoptosis, differentiation, and invasion in several cancers [14]. A previous study reported that overexpression of CRMP4 not only suppressed the invasive ability of prostate cancer cells but also strongly inhibited tumor metastasis in an animal model. They also showed that CRMP4 expression was inversely associated with lymph node metastasis of prostate cancer and validated a new function of CRMP4 as a metastasis suppressor in prostate cancer [15]. A recent study demonstrated that among all CRMPs, CRMP4 was differentially expressed in pancreatic cancer tissues, and CRMP4 knockdown by siRNA reduced venous invasion and liver metastasis [16]. In another recent study analyzing mRNA and protein expression in surgically resected gastric tissues and gastric carcinoma cells, CRMP-4 (Dpysl3) was identified as a potential facilitator of malignant behavior and as an independent prognostic factor in gastric carcinoma. Moreover, the authors also found that CRMP4 mRNA expression levels were positively correlated with some potentially interacting genes in gastric cancer cell lines, such as VEGF, FAK and EZR [17].

Based on these previous reports, we hypothesized that VEGF promotes gastric cancer progression and metastasis by upregulating CRMP4 expression. In the present study, we initially confirmed and validated the mRNA and protein expression of VEGF, VEGFR2 and CRMP4 in gastric cancer tissues. We further investigated the effects on tumor progression of overexpression and knockdown of CRMP4 both *in vitro* and *in vivo*. To our knowledge, this is the first study to characterize CRMP4 expression and its relationship with VEGF, which is significantly associated with a poor prognosis by promoting metastasis.

## RESULTS

### VEGF and CRMP4 proteins and mRNA expression levels are elevated in gastric cancer tissues

**Figure 1:**
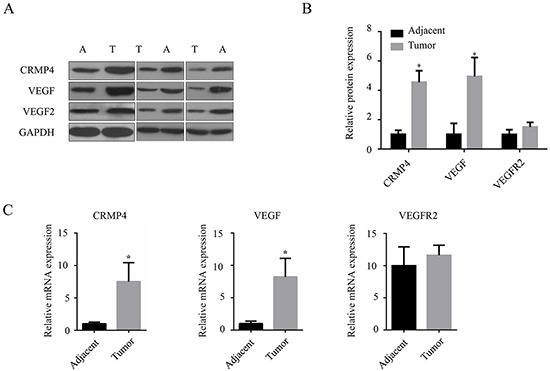
Quantification of VEGF, VEGFR2 and CRMP4 proteins and mRNA levels in gastric cancer tissues **A.** Western blot analysis showing increased expression levels of CRMP4, VEGF and VEGF2. **B.** Relative expression of CRMP4 and VEGF by western blot analysis. **C.** qRT-PCR to detect the relative mRNA expression levels of CRMP4, VEGF and VEGF2 in gastric cancer and tumor-adjacent tissues. All experiments were performed in triplicate. The results from 3 pairs of specimens analyzed by ANOVA are expressed as means ± SD. *P < 0.05 vs tumor-adjacent tissues. GAPDH was used as the control.

VEGF, VEGFR2 and CRMP4 mRNA and protein expression were analyzed in gastric cancer tissues by qRT-PCR and western blot analysis (Figure 1A, 1B and 1C). The mean expression levels of VEGF and CRMP4 were elevated in gastric cancer tissues resected from cancer patients, whereas the expression level of VEGFR2 did not differ significantly between gastric cancer and tumor-adjacent tissues (Figure 1).

**Figure 2:**
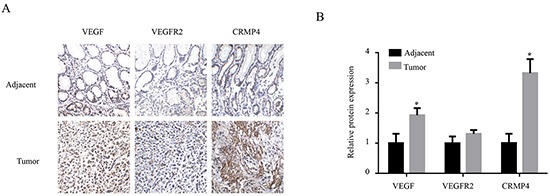
Immunohistochemical analysis of the expression levels of VEGF, VEGFR2 and CRMP4 in tumor sections from gastric cancer patients **A.** Immunohistochemical detection of VEGF, VEGFR2, and CRMP4 expression levels in tumor and tumor-adjacent tissues collected from gastric cancer patients. **B.** The relative intensities of VEGF, VEGFR2 and CRMP4 expression in 10 pairs of tumor and tumor-adjacent tissues were evaluated by IPP based on the IHC results. The results analyzed with ANOVA are expressed as means ± SD. The protein expression levels of VEGF and CRMP4 were significantly increased in gastric cancer tissues compared with tumor-adjacent tissues, whereas the VEGFR2 expression levels did not display any significant increase.

IHC detection of VEGF, VEGFR2 and CRMP4 and their expression levels were performed for 10 pairs of gastric cancer and tumor-adjacent tissue specimens from cancer patients (Figure 2A). The relative protein expression intensity of VEGF and CRMP4 in gastric cancer tissues was evaluated by IPP and compared with the tumor-adjacent tissue (Figure 2A). We observed significantly higher expression levels of VEGF and CRMP4 proteins (*P* < 0.05) in gastric cancer tissues compared with tumor-adjacent tissues (Figure 2B), which was consistent with the results of the western blot analysis. The expression level of VEGFR2 protein did not differ significantly between gastric cancer and tumor-adjacent tissue.

### Prognostic impact of the expression level of CRMP4 in gastric tissues

**Figure 3:**
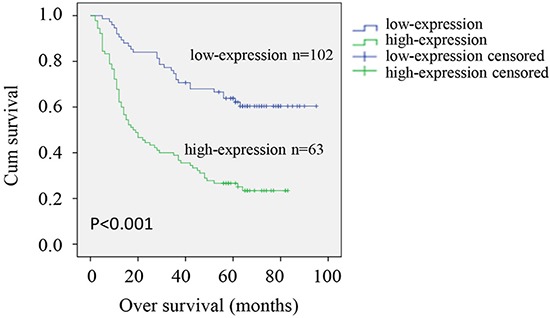
Kaplan–Meier survival analysis of CRMP4 expression in patients with gastric cancer Kaplan–Meier analysis of cumulative survival according to low expression (102 patients) and high expression (63 patients) of CRMP4 determined by IHC in patients with gastric cancer. *P* values are based on the log-rank test. Patients with high CRMP4 expression had a worse survival than those with low expression of CRMP4 (*P* < 0.001).

Correlations between the expression level of CRMP4 and clinicopathological parameters were evaluated in 165 patients with gastric cancer (Table 1). The expression of CRMP4 in gastric tumors did not correlate with gender, age or the size of the primary gastric tumor. However, the expression of CRMP4 in primary gastric tumors was associated with lymph node metastasis, TNM stage and tumor differentiation. Based on the high and low level of CRMP4 expression determined by immunohistochemical analysis, 165 gastric cancer patients were grouped and followed up for survival analysis. As shown in the 8-year survival curve (Figure 3), 165 patients were divided into the CRMP4 high expression (63 patients) and the low expression (102 patients) groups. The high expression group had a significantly shorter median survival time (18 months [95% CI, 10.393-25.607]) than the low expression group (90 months [95% CI, 72.601-107.399]; *P* < 0.001).

**Table 1:** Correlative analysis of CRMP4 expression levels and clinical characteristics of gastric tumors

Clinicalcharacteristics	Casenumber	CRMP4		X^2^	*P*-Value
		high(n, %)	low(n, %)		
Gender				1.124	0.289
Male	104	60(57.7)	44(42.3)		
Female	61	30(49.2)	31(50.8)		
Age				0.542	0.462
≤60	104	59(56.7)	45(43.3)		
>60	61	31(50.8)	30(49.2)		
Lymph node metastasis				7.998	0.005
No	58	23(39.7)	35(60.3)		
Yes	107	67(62.6)	40(37.4)		
TNM stage				10.792	0.013
I	31	11(35.5)	20(64.5)		
II	24	10(41.7)	14(58.3)		
III	50	28(56.0)	22(44.0)		
IV	60	41(68.3)	19(31.7)		
Tumor differentiation				9.000	0.003
High and medium	45	16(35.6)	29(64.4)		
Low and none	120	74(61.7)	46(38.3)		
Tumor size				0.207	0.649
≤4cm	87	46(52.9)	41(47.1)		
>4cm	78	44(56.4)	34(43.6)		

Correlation between CRMP4 expression and the clinical characteristics of gastric cancer patients using the X^2^ test.

### Differential expression of CRMP4, VEGFR2 and VEGF in various gastric carcinoma cell lines

**Figure 4:**
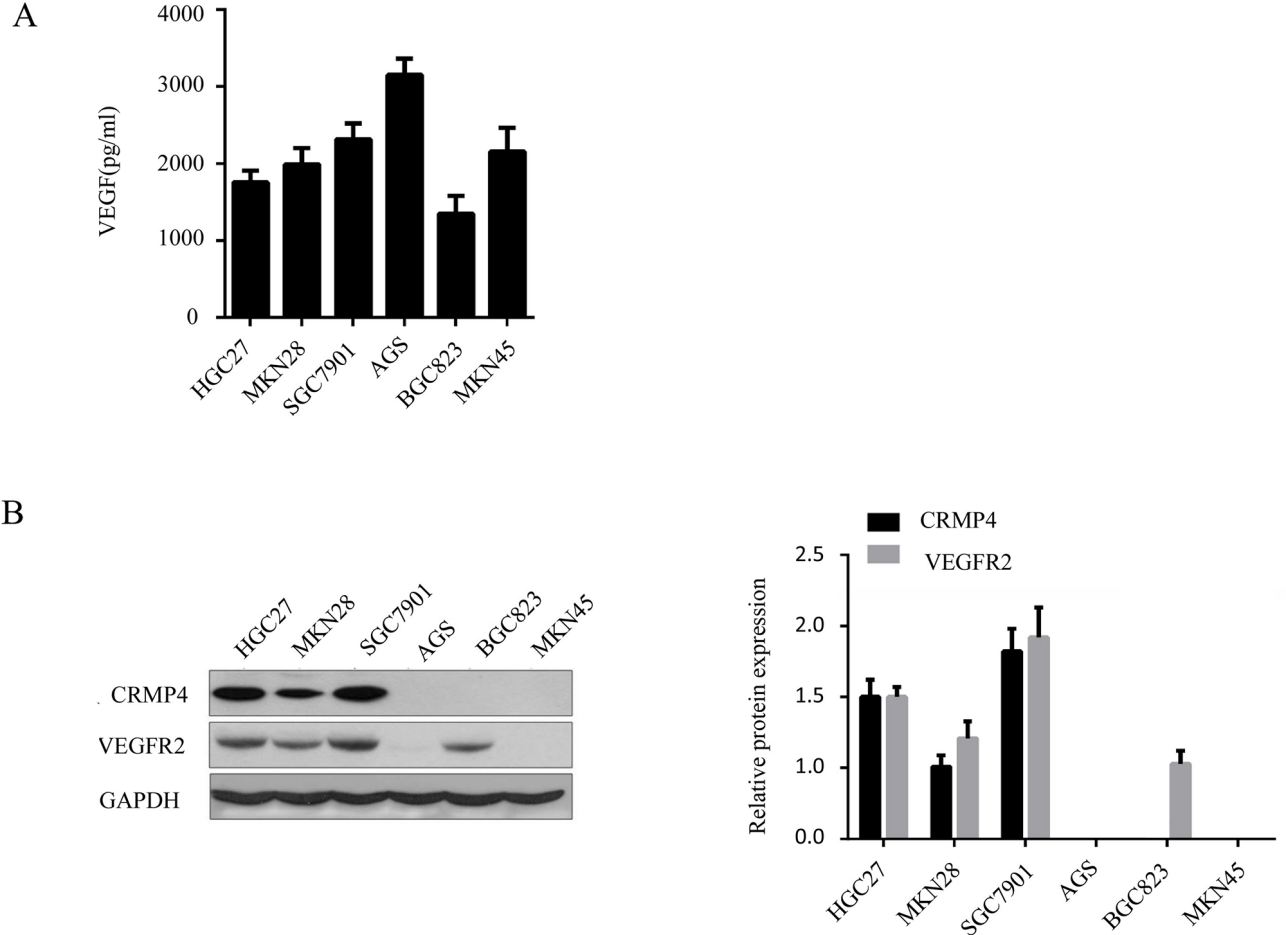
Expression of CRMP4, VEGFR2 and VEGF in various gastric carcinoma cell lines **A.** The expression level of VEGF was quantified by ELISA in six gastric carcinoma cell lines. **B.** Western blot analysis of CRMP4 and VEGFR2 protein expression in each cell line. GADPH was used as a loading control. The expression levels of CRMP4 and VEGFR2 were significantly increased in HGC27 and SGC7901 compared with the other gastric carcinoma cell lines.

To investigate the roles of VEGF and CRMP4 upregulation in the progression of gastric cancer, commonly used gastric cancer cell lines were assessed for VEGFR2 and CRMP4 expression. Endogenous VEGF levels in the cell supernatant were analyzed by ELISA. As shown in Figure 4A, VEGF was detected in the supernatant of various human gastric carcinoma cell lines (HGC27, MKN28, SGC7901, AGS, BGC823 and MKN45). In addition, western blot analysis revealed elevated expression levels of CRMP4 and VEGFR2 inHGC27 and SGC7901 cell lines compared with the other 4 cell lines (Figure 4B). Thus, we selected the HGC27 and SGC7901 cell lines co-expressing both VEGFR2 and CRMP4 for further experiments to study and confirm the role of VEGF upregulation in tumor progression.

### VEGF upregulates CRMP4 expression in the cytoplasm

**Figure 5:**
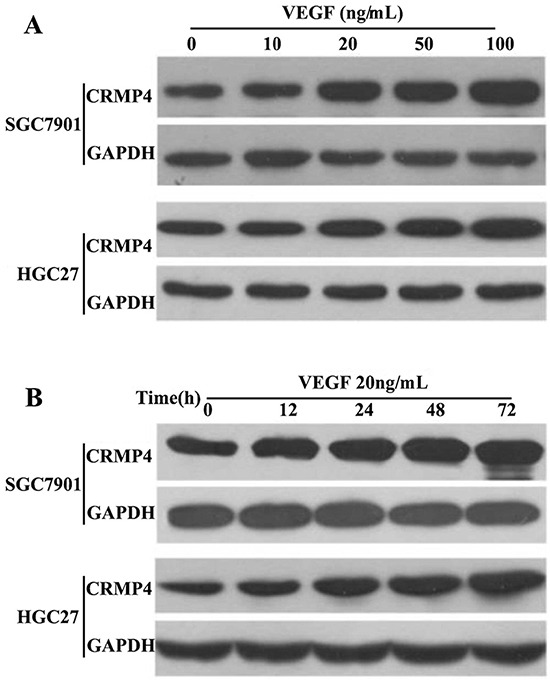
VEGF upregulates CRMP4 expression **A.** CRMP4 expression induced by an increasing concentration of VEGF. SGC7901 and HGC27 gastric cancer cells were treated with different concentrations (0 – 100 ng/mL) of VEGF for 24 hours. **B.** CRMP4 expression induced by increasing incubation times with a constant VEGF concentration of 20 ng/mL. SGC7901 and HGC27 gastric cancer cells were treated with 20 ng/mL of VEGF for various incubation periods ranging from 0 to 72 hours. The CRMP4 protein level was determined by Western blot analysis, and GAPDH served as an internal control. Whole-cell lysates were prepared and immunoblotted using antibodies against CRMP4.

Treatment with VEGF led to a dose-dependent increase in CRMP4 expression in the cytoplasm. Western blot analysis was performed to measure the CRMP4 expression induced by VEGF in the HGC27 and SGC7901 cell lines. We found that VEGF significantly increased the expression of CRMP4 protein in both HGC27 and SGC7901 cells (Figure 5A). Stimulation of HGC27 and SGC7901 cells with 0, 10, 20, 50 and 100 ng/mL VEGF for 24 hours resulted in increased CRMP4 expression. The time courses of VEGF-induced CRMP4 activation was also determined in HGC27 and SGC7901 gastric cancer cells by treating them with 20 ng/mL VEGF for 12, 24, 48 and 72 hours (Figure 5B). A significant increase in CRMP4 expression was observed when the time was increased from 12 to 72 hours in the presence of 20 ng/mL VEGF in both cell lines. Therefore, we optimized the VEGF concentration to 20 ng/mL in further experiments. The maximum expression level of CRMP4 was detected at 48 hours and was significantly higher than the expression levels at 12 hours and 24 hours. Hence, we used this time period in subsequent functional analyses.

### Effects of CRMP4 overexpression and knockdown on cell proliferation, migration and invasion in the SGC7901 and HGC27 cell lines

**Figure 6:**
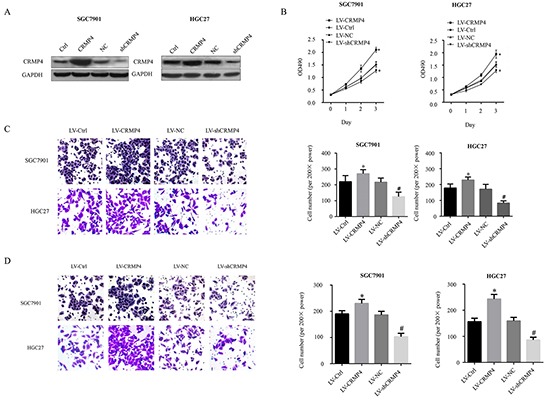
Evaluation of cell proliferation, migration and invasion in SGC7901 and HGC27 cell lines transduced by lentiviral-mediated CRMP4 overexpression and shRNA-mediated knockdown of CRMP4 expression **A.** Western blot results for the expression of CRMP4 in overexpression and knockdown cells. **B.** MTT assay to analyze the proliferation of SGC7901 and HGC27 cell lines transduced by lentiviral-mediated CRMP4 overexpression and shRNA knockdown of CRMP4 expression. **C.** Cell migration of CRMP4 in SGC7901 and HGC27 cell lines transduced with LV-CRMP4, LV-shCRMP4 and LV-Negative controls (LV-NC). **D.** Cell invasion of CRMP4 in SGC7901/HGC27 cell lines transduced with LV-CRMP4, LV-shCRMP4 and LV-NC (*P < 0.01 vs LV-Ctrl, ^#^*P* < 0.01 vs LV-CRMP4.)

The SGC7901 and HGC27 stable cell lines with overexpression and knockdown of CRMP4 were constructed using lentivirus-mediated transduction (Figure 6A). MTT assays were performed to analyze the proliferation of SGC7901 and HGC27 gastric cancer cells. Compared with the control cells, CRMP4-overexpressing cells showed increased proliferation, which was greatly reduced in CRMP4 knockdown cells (Figure 6B).

Consistently, migration and invasion assays showed an increased number of CRMP4-overexpressing cells compared with control cells, whereas the number of CRMP4 knockdown cells was significantly decreased in both SGC7901 and HGC27 cell lines (Figure 6C & 6D).

### The ERK and AKT pathways are involved in the regulation of CRMP4 expression mediated by VEGF

Our findings suggested a potential mechanism by which VEGF regulates CRMP4 expression and further enhances the proliferation, migration and invasion of gastric cancer cells. We identified two potential pathways involving kinases, extracellular-signal-regulated kinase (ERK) and protein kinase B (AKT), the phosphorylation of which is mainly involved in the regulation of VEGF expression in malignant cells. To verify the roles of these signaling pathways in the regulation of VEGF expression in gastric cancer cells, we performed western blot analyses and further evaluated CRMP4 expression after treating both SGC7901/HGC27 cell lines with exogenous VEGF and two independent inhibitors of the MAPK (PD98059, inhibits ERK activity) and PI3K (LY294002, inhibits AKT activity) signaling pathways.

**Figure 7:**
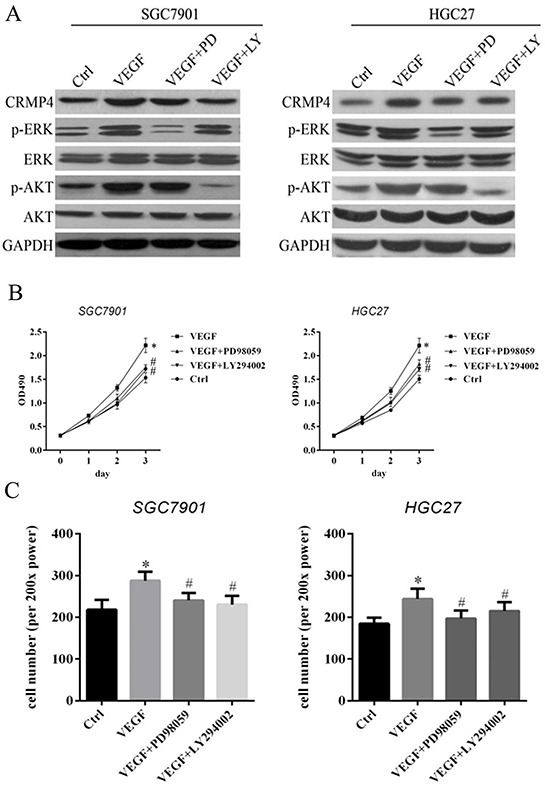
MAPK and PI3K inhibitors (PD98059 and LY294002) contribute to VEGF-mediated CRMP4 expression-induced cell proliferation and migration in SGC7901 and HGC27 cell lines **A.** Western blot analysis of the expression of CRMP4, pERK, ERK, pAKT and AKT in SGC7901 and HGC27 cell lines treated with VEGF, VEGF + PD98059 and VEGF + LY294002. The specific MAPK inhibitor PD98059 effectively suppressed ERK phosphorylation (p-ERK), and the specific PI3K inhibitor LY294002 effectively suppressed AKT phosphorylation (p-AKT). **B.** Cell proliferation assay using the SGC7901 and HGC27 cell lines after treatment with PD98059 and LY294002. A significant reduction in cell proliferation was noticed in the VEGF-treated group compared with the VEGF + inhibitor (PD98059 and LY294002) groups in both the SGC7901 and HGC27 cell lines. **C.** Cell counting assay to analyze migration in SGC7901 and HGC27 cell lines after treatment with VEGF and VEGF inhibitors (PD98059 and LY294002) of the ERK and AKT signaling pathways. (*P < 0.05 vs control, #*P* < 0.05 vs VEGF).

The expression of CRMP4 was increased in the presence of exogenous VEGF in both cell lines compared with normal control cells. However, CRMP4 expression was reduced in both cell lines when the inhibitors (PD98059 or LY294002) were co-administrated with VEGF. Correspondingly, the ERK and AKT pathways were affected by VEGF in gastric cells, as observed by changes in the levels of phosphorylated ERK (pERK) and phosphorylated AKT (pAKT); pERK and pAKT levels were reduced in knockdown cells. In cells treated with VEGF and the inhibitors (Figure 7A), the levels of pERK and pAKT decreased. We further evaluated the roles of the ERK and AKT signaling pathways in the regulation of VEGF-mediated cell proliferation and migration in the SGC7901 and HGC27 cell lines. A statistically significant reduction of proliferation and migration was observed in the VEGF-treated group compared with the VEGF + inhibitor (PD98059 and LY294002) groups in both SGC7901 and HGC27 cell lines (Figure 7B & 7C). These data indicate that suppression of the ERK/AKT signaling pathway contributes to the VEGF-induced reduction of CRMP4 expression and thus can reduce proliferation and metastasis in gastric cancer cells.

### CRMP4 knockdown inhibits tumor growth in gastric cancer *in vivo*

**Figure 8:**
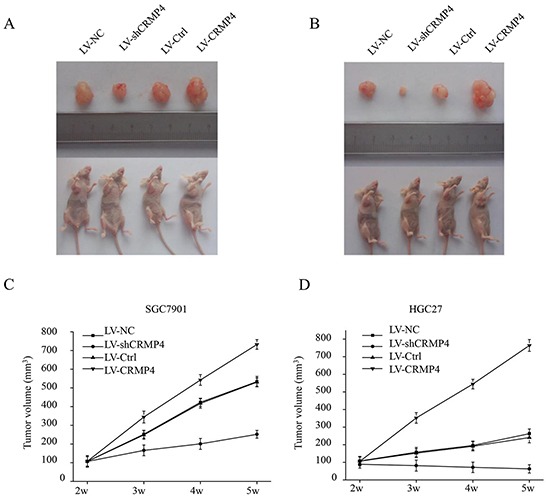
CRMP4 knockdown inhibits tumor growth *in vivo* Images showing examples of xenografted tumor growth *in vivo* at 42 days after injection with LV-CRMP4, LV-shCRMP4 or controls (LV-Ctrl) in SGC7901 **A.** and HGC27 **B.** cells in 4-week-old female BALB/c nu/nu mice. **C.** Statistical analysis of the tumor volume of the xenografted tumor *in vivo* after injection with LV-CRMP4, LV-shCRMP4 or control SGC7901 (C) or HGC27 **D.** cells in 4-week-old female BALB/c nu/nu mice. There was a significant increase in tumor size in the CRMP4 overexpression group and a significant reduction in tumor size in the LV-shCRMP4 group compared with the control groups. (*P < 0.05 vs control).

We further investigated the effects of CRPM4 overexpression and CRMP4 knockdown on the growth of gastric cancer xenograft tumors *in vivo*. The four groups of SGC7901/HGC27 cell lines (LV-Ctrl, LV-sh-CRMP4, LV-NC and LV-CRMP4) were subcutaneously implanted in nude mice. Tumors became palpable from days 20 to 40 and continued to grow. A significant increase and a significant reduction in tumor size were observed in the CRMP4 overexpression group and the LV sh-CRMP4 group, respectively, compared with the control groups (Figure 8A, 8B, 8C and 8D).

## DISCUSSION

CRMP4 has been reported to be involved in the metastatic process of tumor cells [15, 18], and the expression level of CRMP4 in GC tissues may represent a promising biomarker for the malignant behavior of gastric cancer. Expression and functional analyses of CRMP4 in prostate cancer have revealed that CRMP4 is a suppressor of metastasis that is inversely associated with the expression of VEGF [15]. In this study, we validated the mRNA and protein expression of VEGF, VEGFR2 and CRMP4 in gastric cancer tissues by qRT-PCR, immunohistochemical and western blot analyses, and then compared the expression levels of VEGF and CRMP4 with the clinicopathological features of the tumors. Furthermore, we investigated the effect of overexpression and knockdown of CRMP4, both *in vitro* and *in vivo*, on tumor cell proliferation, migration and invasion by constructing stable gastric cell lines using lentiviral-mediated shRNA interference to knock down CRMP4 expression.

The VEGF-VEGFR pathway plays a beneficial role in blood vessel formation, tumor growth and metastasis [19]. A VEGF antibody (bevacizumab) has been shown to reduce the growth of gliomas and to prolong progression-free survival [20, 21]. VEGFR2 can mediate downstream functions of VEGF. In the present study, an increase in VEGF was observed that was accompanied by an increase in CRMP4. It is possible that the VEGF-VEGFR2 pathway mediates the induction of CRMP4. Further studies are needed to determine the mechanisms underlying the interactions among VEGF, VEGFR2 and CRMP4.

Several molecular and signaling pathways are involved in the regulation and inhibition of apoptosis, cell survival and the progression of metastasis in many cancers including gastric cancer. A few recent studies demonstrated that the PI3K/AKT pathway is activated and significantly associated with the development, progression and metastasis of gastric cancer [22–24]. One recent study also indicated that inhibition of the PI3K/Akt pathway suppresses the growth and metastasis of gastric cancer [25]. In another recent study, suppression of the PI3K/Akt pathway using the specific PI3K inhibitor LY294002 showed that PI3K/Akt pathway inactivation affects BMP9-mediated tumor-suppressive effects in gastric cancer cells [26]. Our results support the potential use of LY294002 as an antitumor agent. However, its poor pharmacologic variables of insolubility, a short half-life and liver and skin toxicity should be considered in further clinical applications [27].

Another important pathway, the ERK pathway, plays a regulatory role in cell survival by inhibiting various steps of apoptotic signaling [28–30]. Furthermore, ERK is activated in several tumor cells including prostate cancer cells [31–34]. PD98059 is a potent and selective inhibitor of MAP kinase (also known as MAPK/ERK kinase or MEK kinase) [35]. It inhibitory properties are mediated by binding to the ERK-specific MAP kinase MEK, which prevents the phosphorylation of ERK1/2 by MEK1/2. In another recent study, PD98059 was used to enhance and synergistically potentiate TSA-induced gastric cancer growth arrest and apoptosis by manipulating NF-κB and p21 WAF1/CIP1 independently of Notch, and this procedure was suggested to be a promising treatment strategy for individuals with gastric cancer [36]. However, side effects were observed that were unrelated to the inhibition of MAPK activation by PD98059, and therefore this drug candidate must be used with caution [37, 38].

We investigated two potential pathways, ERK and AKT, and further elucidated a role for the ERK and AKT pathways in VEGF-induced CRMP expression in malignant gastric cancer cells. Our findings demonstrated that suppression of the ERK and AKT signaling pathways contributed to the induction of CRMP4 expression and thus could reduce cell proliferation and metastasis in gastric cancer cells in a manner mediated by VEGF. Whether other pathways contribute to the induction of CRMP4 requires further study.

## CONCLUSION

In conclusion, our results provide evidence for the involvement of CRMP4/VEGF and the role of the ERK and AKT signaling pathways in gastric tumor progression and metastasis. Inhibition of the ERK and AKT signaling pathways using MAPK or PI3K inhibitors resulted in reduced proliferation and metastasis in gastric cancer cells *in vitro* and *in vivo*. This novel study highlights potential therapeutic applications for the treatment of gastric cancer.

## MATERIALS AND METHODS

### Cells and cell cultures

The human gastric carcinoma cell lines, HGC27, MKN28, SGC7901, AGS, BGC823 and MKN45 (CCTCC, China), were maintained in DMEM supplemented with 10% (v/v) fetal bovine serum (FBS) (Gibco BRL, Gaithersburg, MD) and incubated at 37°C in a humidified incubator with 5% CO_2_. To maintain uniform conditions, all experiments were conducted using cell passages 4-6.

### Clinical data and tumor specimen collection from patients with gastric cancer

A total of 165 gastric cancer patients who were enrolled and admitted for treatment at the gastrointestinal surgery unit of The First Affiliated Hospital of Sun Yat-sen from January 2006 to December were included in our study. The inclusion criteria were as follows: 1, a clear diagnosis of gastric cancer during surgery in parallel; 2, preoperative neoadjuvant chemotherapy. Patients with a simultaneous diagnosis of multiple cancers were excluded from the study. After obtaining their written informed consent, pathology specimens were collected from all 165 patients by surgical excision of tumors from each patient, which included cancerous tissues and adjacent tissues (without cancer invasion).

### Plasmid construction and transfection

To construct stable CRMP4-overexpressing cells, full-length human CRMP4 cDNA (NCBI Reference Sequence: NM_001387.2) was cloned into the HpaI/XhoI restriction sites of LV-004 (Forevergen, China) according to the manufacturer's instructions. Lentivirus-mediated shRNA was used to knock down CRMP4 expression. The vector LV-008 (Forevergen, Guangzhou) with a U6 promoter was used to generate small hairpin RNAs. The small hairpin RNA of CRMP4 (shCRMP) (sense 5′-AACTGGACAACTTCACAGCCATTTTCA AGAGAAATGGCTGTGAAGTTGTCCTTTTTT C-3′) was sub-cloned into the HpaI/XhoI restriction sites. LV-008, LV-004 and packaging vectors were co-transfected into HEK 293T cells. At 48 and 72 hours post-transfection, the supernatant was collected. Lentiviruses were recovered after ultracentrifugation for 1.5 hours at 25,000 rpm and re-suspended in PBS. The lentivirus infections were performed in the presence of 5-10 μg/mL polybrene. The cells were cultured in medium for 48 hours with 2 μg/mL puromycin for 2 weeks to generate stable cell lines. A commercial negative control (NC) was used.

### Immunohistochemistry (IHC) analysis

Immunohistochemistry was performed using a polymer-based technology (Envision; Dako) as described previously [39]. Gastric cancer and adjacent tissue specimens were resected and washed in PBS. Specimens were fixed with 4% paraformaldehyde overnight at 4°C and then embedded in paraffin, cut to a size of 4 mm using a microtome and fixed onto the slide. The tissue sections were dewaxed in xylene and rehydrated using graded alcohol concentrations according to standard procedures. The sections were subsequently submerged in EDTA (pH 8.0) and autoclaved at 121°C for 5 min to retrieve antigenicity. After washing in phosphate-buffered saline (PBS, 0.1 M, PH 7.4, 3 times for 5 min), endogenous peroxidase was blocked by incubation in 3% hydrogen peroxide for 15 min at room temperature. After rinsing with PBS, the slides were incubated for 1 hour at room temperature with primary antibodies. The primary antibodies and dilutions were as follows: VEGF (Abcam, USA), 1:100 ; VEGFR2 (CST, USA), 1:100 dilution; CRMP4 (VEGF (Abcam, USA), 1:100 dilution.

Immunostaining was performed using the Envision system with diaminobenzidine (Dako Cytomation, Glostrup, Denmark). Negative controls were treated with PBS instead of primary antibodies. The relative intensity of VEGF, VEGFR2 and CRMP4 protein expression was evaluated using Image-Pro Plus (IPP version 6.0; Media Cybernetics, Silver Spring, Maryland, USA). For each section, 10 digital images were captured at a resolution of 1360 × 1024 pixels and magnification of 400× using a BX51WI microscope (Olympus). The measurement parameters included the density mean, area sum and integral optical density (IOD). The optical density was calibrated, the image was converted to gray scale and the values were counted.

For the survival analysis, semi-quantitative analysis of CRMP4 expression was performed by examining the IHC slides of the gastric cancer patients. The expression of CRMP4 was assessed independently by two pathologists who were blinded to the clinical data based on the proportion of CRMP4-positive cells. The score was assigned using a 4-point scale: 0, ≤ 5% positive tumor cells; 1, 5% > and ≤ 25% positive tumor cells; 2, 25% > and ≤ 50% positive tumor cells; 3, 50% > and ≤ 75% positive tumor cells; 4, > 75% positive tumor cells. A score equal to or higher than 6 was defined as high expression, while a score below 6 was considered low expression.

### Quantitative real-time PCR (qRT-PCR)

Gastric cancer and adjacent tissue specimens were washed with cold PBS quickly after surgical resection and stored at −80°C. Total RNA was extracted using TRIzol Reagent (Invitrogen, Carlsbad, CA), and reverse transcription was performed using an Advantage HRT for PCR Kit (Clontech, Mountain View, CA) according the manufacturer's instructions. For qPCR analysis, aliquots of double-stranded cDNA were amplified with primers (as specified below) using a SYBR Green PCR Kit (Applied Biosystems, Carlsbad, CA) and an ABI PRISM 7900 Sequence Detector. The threshold cycle (CT) was measured during the exponential amplification phase, and the amplification plots were analyzed using SDS 1.9.1 software (Applied Biosystems). qRT-PCR to evaluate VEGFR2 and CRMP4 expression in gastric cancer cell lines was performed using the following primers: VEGFR2, 5′- CACCACTCAAACGCTGACATGTA -3′ (sense) and 5′- GCTCGTTGGCGCACTCTT-3′ (antisense); CRMP4, 5′- AAACCCGCATGTTGGAAATGG-3′ (sense) and 5′- TGACCTTTGTGACGTAGAGAGG-3′ (antisense); human GAPDH, 5′-ACCCATCACCATCTTCCAGGAG-3′ (sense) and 5′-GAAGGGGCGGAGATGATGAC-3′ (antisense). The relative gene expression levels were calculated using the comparative Ct (ΔΔCt) method, where the relative expression is calculated as 2^−ΔΔCt^, and Ct represents the threshold cycle.

### Western blot

Gastric cancer and adjacent tissue specimens were washed with cold PBS quickly after resection and stored at −80°C for subsequent analysis. Whole cell lysates from gastric cancer cells were harvested with cell lysis buffer using the NucBuster™ Protein Extraction Kit (Novagen, Germany) according to the manufacturer's instructions. Western blot analyses were performed using the standard protocol with antibodies against VEGF (Abcam, USA) 1:1000 dilution, VEGFR2 (CST, USA) 1:1000 dilution, CRMP4 (Abcam, USA) 1:1000 dilution, pERK (CST, USA) 1:1000 dilution, ERK (CST, USA) 1:1000 dilution, pAKT (CST, USA) 1:1000 dilution, and AKT (CST, USA) 1:1000 dilution. GADPH (CST, USA) at a dilutions of 1:1000 was used as a control.

### Enzyme-linked immunosorbent assay (ELISA)

Gastric cells were seeded into 96-well plate at a density of 10^4^ cells/well. At 24 hours, the cells were collected, and ELISA was performed to assess the content of VEGF according to the manufacturer's instructions. ELISA kits to detect VEGF were purchased from R&D (Abingdon, England).

### Proliferation assay

Cell viability was measured using the quantitative colorimetric 3-[4,5-dimethylthiazol-2- yl]-2,5-diphenyl-tetrazolium bromide (MTT) assay [40]. Cells were cultured overnight in a 24-well plate at a density of 4 × 10^4^ cells/mL. The cells were then incubated in DMEM containing 0.5 mg/mL MTT for 2 hours at 37°C. The formazan in viable cells was dissolved in dimethylsulfoxide and measured by reading the optical densities using a microplate reader (DYNEX Technologies Inc., Chantilly, VA, USA) at an absorption wavelength of 570 nm.

### *In vitro* migration and invasion assays

The migration and invasion assays were performed as previously described [41, 42]. Cells were seeded in triplicate into the upper compartment of a chamber (Transwell, Costar, Cambridge, MA) at a density of 2.5 × 10^4^ cells/50 μL per well and supplemented with serum-free DMEM. The lower compartment was filled with 30 μL of DMEM containing 10% fetal bovine serum (FBS) serum. The cells could migrate through a membrane with a pore size of 8 μm and a Matrigel-coated membrane (Becton Dickinson, Bedford, MA) (for the migration assay and the invasion assay, respectively). After 48 hours in a humidified atmosphere containing 5% CO_2_ at 37°C, the cells in the upper compartment were removed and the migrated cells in the lower compartment were fixed in absolute methanol and stained with 10% Giemsa solution (Sigma, Schnelldorf, Germany). Finally, the fixed cells were photographed using a microscope with a digital imaging system and counted as the mean ± standard deviation (SD) per filter under five high-power fields.

### Mouse xenograft experiments

BALB/c nu/nu mice were purchased from Shanghai Slac Laboratory Animal Co., Ltd. (Shanghai, China). The animal experiments were approved by The Institute Research Medical Ethics Committee of Sun Yat-Sen University. The mice were fed sterilized drinking water and food, and povidone-iodine was used to disinfect the vaccination sites. Four-week-old female BALB/c nu/nu mice were used to develop a mouse model by inoculating the right armpit of the nude mice with LV-CRMP4, LV-shCRMP4 and control HGC27 and SGC7901 cells. The tumor volume was measured once a week (volume (mm^3^) = length × width^2^ × 0.5).
